# Enterolith Causing Afferent Loop Perforation After Distal Gastrectomy

**DOI:** 10.7759/cureus.37021

**Published:** 2023-04-01

**Authors:** Naoya Ozawa, Masaki Kanzaki

**Affiliations:** 1 General Surgery, Tokyo Bay Urayasu Ichikawa Medical Center, Urayasu, JPN

**Keywords:** roux-en-y reconstruction, gastrectomy, obstruction, perforation, enterolith, afferent loop syndrome

## Abstract

Afferent loop syndrome is a complication that occurs after the Billroth Ⅱ reconstruction or Roux-en-Y reconstruction and can also be caused by enteroliths. We experienced a case of duodenal perforation due to afferent loop syndrome caused by an enterolith, in which surgical removal of the enterolith and decompression of the duodenum were effective. A 73-year-old female who underwent distal gastrectomy and Roux-en-Y reconstruction for gastric cancer 14 years ago came to the hospital with acute abdominal pain and underwent emergency surgery for afferent loop syndrome and duodenal perforation due to enterolith. The patient underwent removal of the enterolith, drain placement, and placement of a decompression tube in the duodenum. Postoperatively, percutaneous drainage of the intra-abdominal abscess was necessary, but the patient was saved without reoperation. Afferent loop perforation may occur with obstruction due to enteroliths, and the surgical insertion of a tube to decompress the afferent loop is effective.

## Introduction

Afferent loop syndrome is a relatively rare complication of Billroth Ⅱ reconstruction or Roux-en-Y reconstruction after gastrectomy. Afferent loop syndrome is commonly caused by adhesions, intussusception, malignancy, and internal hernia [1,2], but can also be caused by enteroliths [3-6]. However, the rate of afferent loop syndrome due to enterolith is unknown.

Afferent loop may perforate when ischemia or necrosis occurs due to obstruction [7], although this is a rare condition. No case of afferent loop perforation associated with enterolith was reported in the literature. Surgery is often performed for afferent loop syndrome, although endoscopic procedures have recently been used [8]. The most effective treatment for afferent loop syndrome has not been established. In this report, we describe a case of perforation of the duodenum due to afferent loop syndrome caused by an enterolith, which led to pan-peritonitis, but the patient was successfully saved by surgery.

## Case presentation

**Figure 1:**
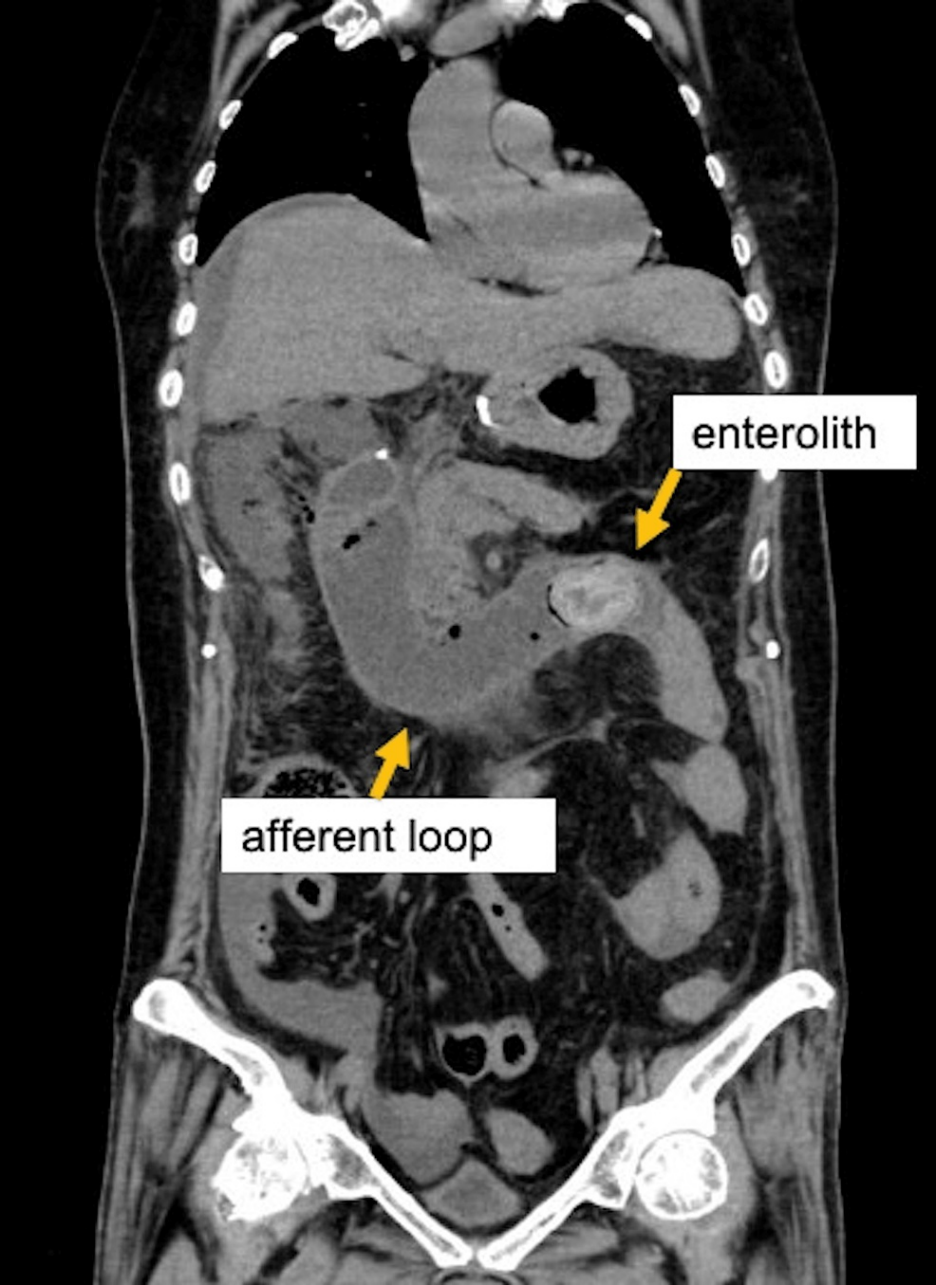
The afferent loop is obstructed by the enterolith and dilated. Extraluminal air and ascitic fluid are present around the duodenum.

A 73-year-old female presented to the emergency department with acute epigastric pain and a two-week history of anorexia. She had diabetes and hypertension and had previously undergone cholecystectomy for gallstones. Fourteen years prior, she underwent distal gastrectomy and Roux-en-Y reconstruction for stomach cancer. Vital signs were blood pressure of 97/41 mmHg, heart rate of 126 beats/min, respiration rate of 46 breaths/min, and body temperature of 36.0 ℃. On physical examination, her abdomen was hard, diffusely tender, and percussion tenderness. Laboratory tests showed leukocyte count of 19,700/mm^3^ and acidosis (pH of 7.124, partial pressure of carbon dioxide (PCO2) of 40.4 mmHg, bicarbonate (HCO3-) of 12.7 mmol/L, lactate of 135 mg/dl). Computed tomography (CT) revealed afferent loop obstruction with an enterolith, periduodenal free air, and free intraperitoneal fluid (Figure 1); therefore, we made a diagnosis of pan-peritonitis due to afferent loop perforation and performed emergency surgery. 

**Figure 2:**
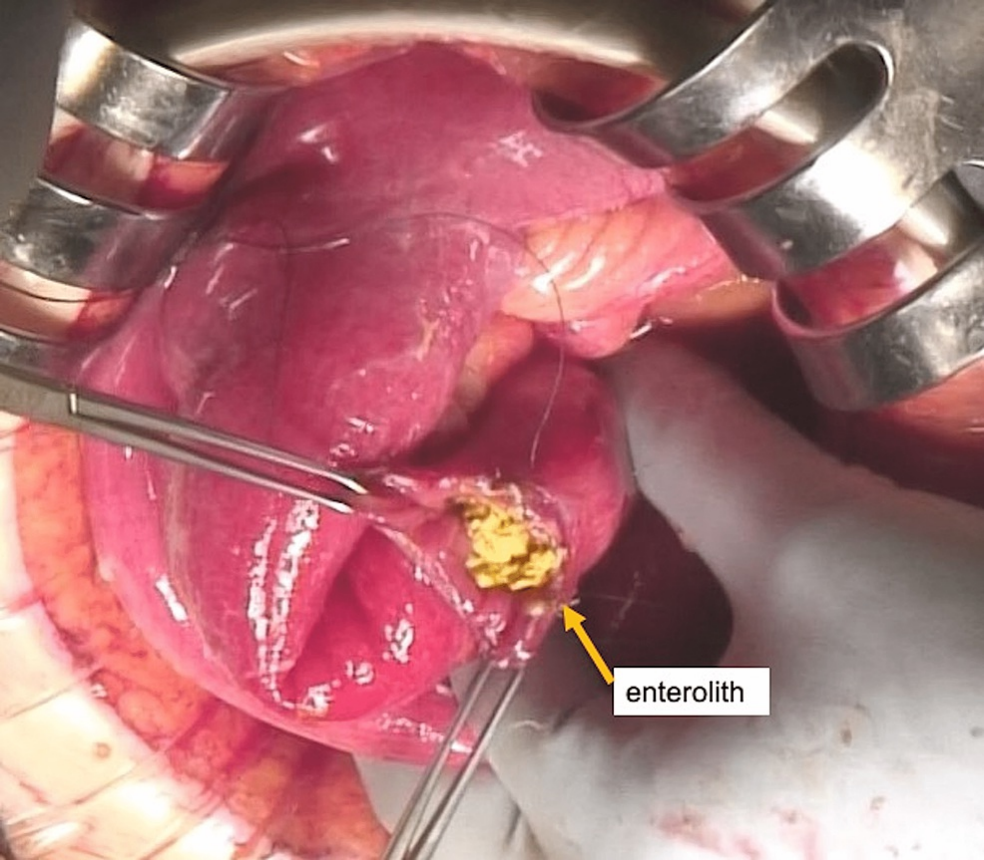
Enterolith was removed through an incision in the afferent loop.

There was a large amount of ascites in the abdominal cavity and a hard mass was palpated in the jejunum of the afferent loop. The incision of the afferent loop revealed an enterolith (Figure 2). The enterolith was removed and the incision was sutured. There was a hole in the right paracolic gutter, through which bile was found to be draining. Intestinal fluid flowing from the perforated duodenum was expected to leak from the retroperitoneum into the abdominal cavity through this hole. The site of duodenal perforation could not be identified because the Cattell-Braasch maneuver and Kocher maneuver were not possible due to adhesions around the duodenum. It was difficult to close the hole in the paracolic gutter, so a drain was placed near the hole. We determined that decompression within the afferent loop was necessary to reduce the amount of intestinal fluid draining from the perforation, so a tube was inserted into the afferent loop and drained out of the body. 

The patient was admitted to the intensive care unit and transferred to the general ward three days after surgery. On postoperative day nine, a CT scan showed ascites accumulation in the right and left abdomen and pelvis; on day 12, the accumulation in the right abdomen was percutaneously punctured and an abscess drained. We performed a duodenal contrast study on day 14 using a tube inserted into the afferent loop. A small amount of intestinal fluid leaked from the duodenum, but it was determined that it could be drained through the right paracolic drain. The paracolic drain was removed as there was no more drainage. The tube inserted into the afferent loop was removed after confirming that it was safe to clamp. A CT taken on day 22 to investigate the cause of the fever revealed a residual abscess in the pouch of Douglas, which was punctured and drained transvaginally. Her subsequent course was good, but negative pressure wound therapy of the midline incision and rehabilitation took time, and she was discharged on day 62.

## Discussion

This report shows that there is a case of duodenal perforation with afferent loop syndrome caused by enterolith, and a tube insertion to decompress the afferent loop is effective. 

Afferent loop syndrome is a rare complication that can occur with the Billroth Ⅱ reconstruction or Roux-en-Y reconstruction after gastrectomy. The incidence of afferent loop syndrome in Roux-en-Y reconstruction after distal gastrectomy is reported to be 0.2% [9]. Afferent loop syndrome is commonly caused by adhesions, intussusception, malignancy, and internal hernia [1,2], it is also caused by enterolith, but is very rare [5,7]. The rate of afferent loop syndrome caused by enteroliths has not been reported. In acute afferent loop syndrome, complete obstruction of the afferent loop leads to serious complications such as ischemia and necrosis, which progresses to perforation and peritonitis [7]. There is a case report of afferent loop perforation due to obstruction which was caused by pressure exerted by the mesentery of the distal jejunal loop after Billroth Ⅱ reconstruction [10], but this is a rare condition. Cases of afferent loop syndrome caused by enterolith, resulting in cholangitis and pancreatitis, have been reported [3-6]. However, there have been no reported cases of afferent loop perforation due to obstruction caused by enterolith. This is the first case to be reported in the literature.

Tube decompression of the afferent loop is effective as an approach to its perforation. Since little intestinal fluid flowed out of the drain in the paracolic gutter postoperatively and drainage subsequently ceased, the perforation was considered to have closed spontaneously. In this case, the decompression of the tube inserted into the duodenum contributed to the closure of the perforation. 

In addition, contrast studies of the afferent loop are useful in the evaluation of obstruction or perforation. In this case, a contrast study of the afferent loop was performed through the tube to confirm that the obstruction had been released and that there was no significant leakage of intestinal fluid from the afferent loop. Contrast is useful to evaluate the degree of obstruction, the closure of the perforation, and if the fistula becomes an external fissure. There is a case report of percutaneous puncture of a dilated afferent loop for decompression before surgery [3], so we think the insertion of a drainage tube into the afferent loop may be useful in afferent loop obstruction. Percutaneous transhepatic biliary drainage is reported to be useful when the intrahepatic bile duct is dilated due to obstruction of the afferent loop [6].

## Conclusions

Afferent loop perforation may occur with afferent loop obstruction due to enteroliths, and the surgical insertion of a tube to decompress the afferent loop is effective. Since perforation of the afferent loop is rare, the appropriate surgical strategy has not yet been determined, and further studies are needed.
